# Celiac disease gut microbiome studies in the third millennium: reviewing the findings and gaps of available literature

**DOI:** 10.3389/fmedt.2024.1413637

**Published:** 2024-09-16

**Authors:** Vanessa C. C. Luz, Sónia Gonçalves Pereira

**Affiliations:** Center for Innovative Care and Health Technology, School of Health Sciences, Polytechnic of Leiria, Leiria, Portugal

**Keywords:** celiac disease, gut microbiome, gut microbiota, autoimmunity, next-generation sequencing, PRISMA-ScR

## Abstract

Celiac disease is an autoimmune enteropathy caused by the ingestion of minute amounts of gluten in a subset of genetically predisposed individuals. Its onset occurs at different ages and with variable symptoms. The gut microbiome may contribute to this variability. This review aims to provide an overview of the available research on celiac disease gut microbiome and identify the knowledge gap that could guide future studies. Following the guidelines of the Preferred Reporting Items for Systematic Reviews and Meta-analysis extension for Scoping Reviews (PRISMA-ScR), four electronic databases were searched for literature from January 2000 to July 2023 addressing celiac disease gut microbiome characterization using next-generation sequencing (NGS) approaches. From the 489 publications retrieved, 48 publications were selected and analyzed, focusing on sample characterization (patients, controls, and tissues) and methodologies used for NGS microbiome analysis and characterization. The majority of the selected publications regarded children and adults, and four were randomized clinical trials. The number of participants per study greatly varied and was typically low. Feces were the most frequently tested sample matrix, and duodenal samples were analyzed in one-third of the studies. Incomplete and diverse information on the methodological approaches and gut microbiome results was broadly observed. While similar trends regarding the relative abundance of some phyla, such as Pseudomonadota (former Proteobacteria), were detected in some studies, others contradicted those results. The observed high variability of technical approaches and possibly low power and sample sizes may prevent reaching a consensus on celiac disease gut microbiome composition. Standardization of research protocols to allow reproducibility and comparability is required, as interdisciplinary collaborations to further data analysis, interpretation, and, more importantly, health outcome prediction or improvement.

## 1 Introduction

Celiac disease (CeD) is an immune-mediated condition caused by the ingestion of gluten (a complex mixture of proteins found in wheat, barley, or rye) in nearly 3% of genetically predisposed individuals carrying the human leucocyte antigen (HLA) DQ2 and/or DQ8 haplotypes (1). The only available treatment for CeD patients is a gluten-free diet (GFD), which gradually leads to intestinal mucosa healing and symptom control. Nevertheless, 30%–40% of patients continue to experience symptoms and persistent enteropathy despite adhering to a GFD (2). This conundrum suggests the involvement of other factors in CeD triggering and maintenance, highlighting that genetic, autoimmune, and environmental factors are necessary but insufficient for CeD development (3).

The gut microbiome is a complex and diverse ecosystem that plays an important role in intestinal homeostasis and overall health. Although the gut microbiome is composed of all microorganisms (archaea, bacteria, fungi, viruses), current research is mainly focused on bacteria. Gut bacteria contribute to host protection against pathogens and have a major role in modulating the host's immune system, including T and B lymphocyte activation, by producing a variety of molecules that interact with it (4–6). Several studies addressing CeD–gut microbiome interplay have been conducted over the last years, with dysbiosis, defined as an unbalanced microbiome, acknowledged as a factor contributing to the loss of gluten tolerance (6). However, interpreting how and which microbiome-specific components participate in CeD triggering and maintenance is yet fairly unknown.

This review aimed to synthesize the available research on the interplay between CeD and gut microbiome, using next-generation sequencing (NGS) approaches, and identify the findings and current gaps in CeD–gut microbiome research. Delving into the methodologies used for NGS microbiome analysis, identifying the assessed CeD populations (pediatric vs. adult, active vs. remission, treated vs. undiagnosed) and samples (duodenal, fecal, others), and assessing the significant microbiome findings are important to determine which questions are left to investigate and which methodologies are more adequate. Another goal is to highlight the latest findings regarding the impact of gut microbiome on CeD development, providing an overview of the existing research.

## 2 Methods

The compilation of the information used in this review was conducted according to PRISMA-ScR (Preferred Reporting Items for Systematic Reviews and Meta-analysis extension for Scoping Reviews) guidelines (7).

### 2.1 Eligibility criteria

To be included in this review, publications should focus on the CeD population and gut microbiome or microbiota. Studies should be available online and published between January 2000 and July 2023, involving human participants and focusing on gut microbiome characterization by NGS. We included studies performed in patients with a confirmed diagnosis of CeD or newborns and children at risk of developing CeD who later received a confirmed diagnosis. No restriction on age, gender, ethnicity, geography, duration of illness, and treatment status was considered.

Publications were excluded if they employed a two-step gut microbiome characterization performed by culture-based approaches followed by NGS. Studies using other molecular biology approaches, such as DGGE or similar, were also excluded.

### 2.2 Information sources and search

We performed an extensive literature search across four databases, namely, Google Scholar, PubMed, Scopus, and Web of Science. We included original peer-reviewed articles (randomized controlled trials, observational studies, and letters to the editor), commentaries, protocols, guidelines, and recommendations published in any language, as well as gray literature including conference proceedings, conference posters, workshops, briefings, government documents and reports, policy statements, theses, and dissertations published elsewhere.

The search strategy was previously debated among the authors and their collaborators to obtain the best collection of keywords. The conjunctive Boolean operators (e.g., AND, OR, NOT) were selected to combine and focus on the desired search results (Table 1). The databases were searched from July to August 2023, and all available studies addressing the research topic were retrieved for analysis.

**Table 1:** Data collection procedures. Number of publications and search strategy used on the literature databases: Google Scholar, PubMed, Scopus, and Web of Science.

Database	Publications	Search strategy
Google Scholar	149	Allintitle: microbiota “celiac disease"
PubMed	52	((“celiac disease"[mesh Terms] OR “celiac disease"[Title/Abstract] OR “coeliac disease"[Title/Abstract] OR “celiac sprue"[Title/Abstract] OR “gluten sensitivity"[Title/Abstract] OR “gluten intolerance"[Title/Abstract]) AND (“intestines"[mesh Terms] OR “Gut"[Title/Abstract] OR “gastrointestinal"[Title/Abstract] OR “duodenum"[Title/Abstract] OR “bowel"[Title/Abstract] OR “feces"[Title/Abstract] OR “small intestine"[Title/Abstract]) AND (“sequencing"[Title/Abstract] OR “next-generation"[Title/Abstract] OR “culture"[Title/Abstract] OR “culturomics"[Title/Abstract]) AND (“microbiota"[mesh Terms] OR “microflora"[Title/Abstract] OR “bacteria"[Title/Abstract] OR “flora"[Title/Abstract] OR “microbiome"[Title/Abstract] OR “commensal"[Title/Abstract])) NOT “review"[Publication Type]
Scopus	194	TITLE-ABS (“celiac disease”) OR TITLE-ABS (“coeliac disease”) OR TITLE-ABS (“celiac sprue”) OR TITLE-ABS (“gluten sensitivity”) OR TITLE-ABS (“gluten intolerance”) AND TITLE-ABS (gut) OR TITLE-ABS (gastrointestinal) OR TITLE-ABS (duodenum) OR TITLE-ABS (bowel) OR TITLE-ABS (feces) OR TITLE-ABS (small AND intestine) AND TITLE-ABS (next-generation) OR TITLE-ABS (culture) OR TITLE-ABS (culturomics) AND TITLE-ABS (microbiota) OR TITLE-ABS (microflora) OR TITLE-ABS (bacteria) OR TITLE-ABS (flora) OR TITLE-ABS (microbiome) OR TITLE-ABS (commensal)
Web of Science	94	(celiac OR coeliac) AND (sequencing OR next-generation) AND (microbiome OR microbiota) AND (gut OR gastrointestinal OR intestinal OR feces OR feces)

### 2.3 Data selection and evidence analysis

Data were collected in a form created in an Excel sheet. Titles and abstracts were reviewed by 2 independent reviewers, and eligibility criteria were applied. Studies that did not comply with the review subject were excluded. Articles that were unavailable online were also excluded as those that were duplicated between the different databases. Divergences between reviewers were resolved in discussion sessions.

After selection, the qualifying full-text publications were critically reviewed using the reference management software Zotero, and a detailed analysis of the results was performed. From the included studies, an evidence table was created, containing the following information: authors; title; journal; year; country; type of study; study design; aims; inclusion and exclusion criteria; methodology; conclusions; number and characteristics of participants and controls; their genetic predisposition status and clinical features; type of samples; measured outcomes; technology used to extract and sequence the microbiome; technology used to analyze the microbiome; methods and software used to analyze and interpret the microbiome elements such as amplicon sequence variants (ASV) or operational taxonomic units (OTU), abundance, and richness/diversity; and also the techniques used to perform the statistical analysis. Lastly, the table also contained information regarding microbiome composition results, as to the presence/absence/brief information of the former phyla Firmicutes (currently Bacillota), Bacteroidetes (currently Bacteroidota), Fusobacteria (currently Fusobacteriota), Proteobacteria (currently Pseudomonadota), Verrucomicrobia (currently Verrucomicrobiota), and Actinobacteria (currently Actinomycetota) and the genera Bacteroides and Lactobacillus, as those were the most frequently mentioned and discussed phylogenetic groups in the reviewed studies. Data extraction was conducted by one reviewer, and accuracy was confirmed by a second reviewer.

The microbial taxonomy nomenclature used in this scoping review took into consideration the nomenclature used in the reviewed studies, not the recent updates on microbial taxonomy since the reviewed manuscripts are prior to these updates. The recent nomenclature can be seen in the abstract and in the titles under the Results section.

## 3 Results

### 3.1 Description of the selected studies

**Figure 1:**
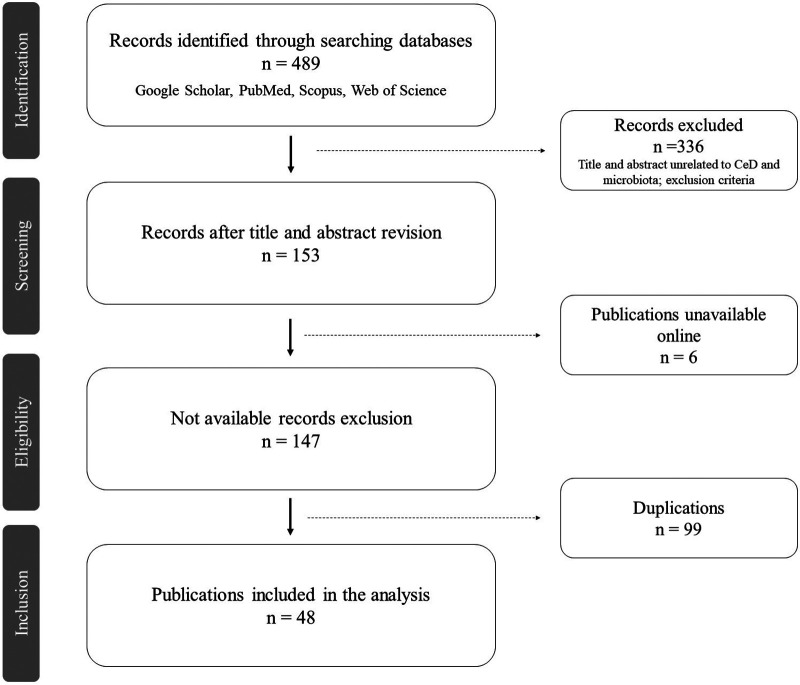
PRISMA flow diagram (7). Workflow of current review: identification, search on multiple databases (Google Scholar, PubMed, Scopus, Web of Science); screening, reviewing the titles and abstracts of retrieved publications and excluding those unrelated to the review theme; eligibility, retaining publications of interest after excluding those unavailable online; inclusion, including the selected publications after removing the duplicates, thus reaching the final number of articles analyzed in this review.

A total of 489 articles were found during the literature search, 149 from Google Scholar, 52 from PubMed, 194 from Scopus, and 94 from Web of Science (Table 1). After assessing the title and abstract, 336 publications were excluded, as well as 97 duplicated publications and 6 that were unavailable. Fifty full-text publications were assessed, 1 conference abstract and 1 workshop abstract were also excluded as the content, and results were published in articles included in this review, raising the number of duplications to 99 after the full analysis (Figure 1). A total of 48 publications were retained, comprising 40 original articles, 2 abstracts (8, 9), 3 posters (10–12), 1 academic dissertation (13), 1 conference abstract (14), and 1 briefing (15).

Among the included original publications, 21 were prospective, 23 were cross-sectional, 2 were both prospective and cross-sectional (16, 17), and 1 poster abstract did not mention this information (11). Of note, 4 of the 21 prospective studies were randomized controlled trials (RCT), focused on evaluating the effect of a 3-month *Bifidobacterium breve* strain food supplement (18), probiotic mixture (19), *B. infantis* “Natren Life Start (NLS) super strain” supplement (20), and registered gluten-free bread on the gut microbiome of CeD patients (21). The first study involved children as their study population and adults in the other three studies.

**Figure 2:**
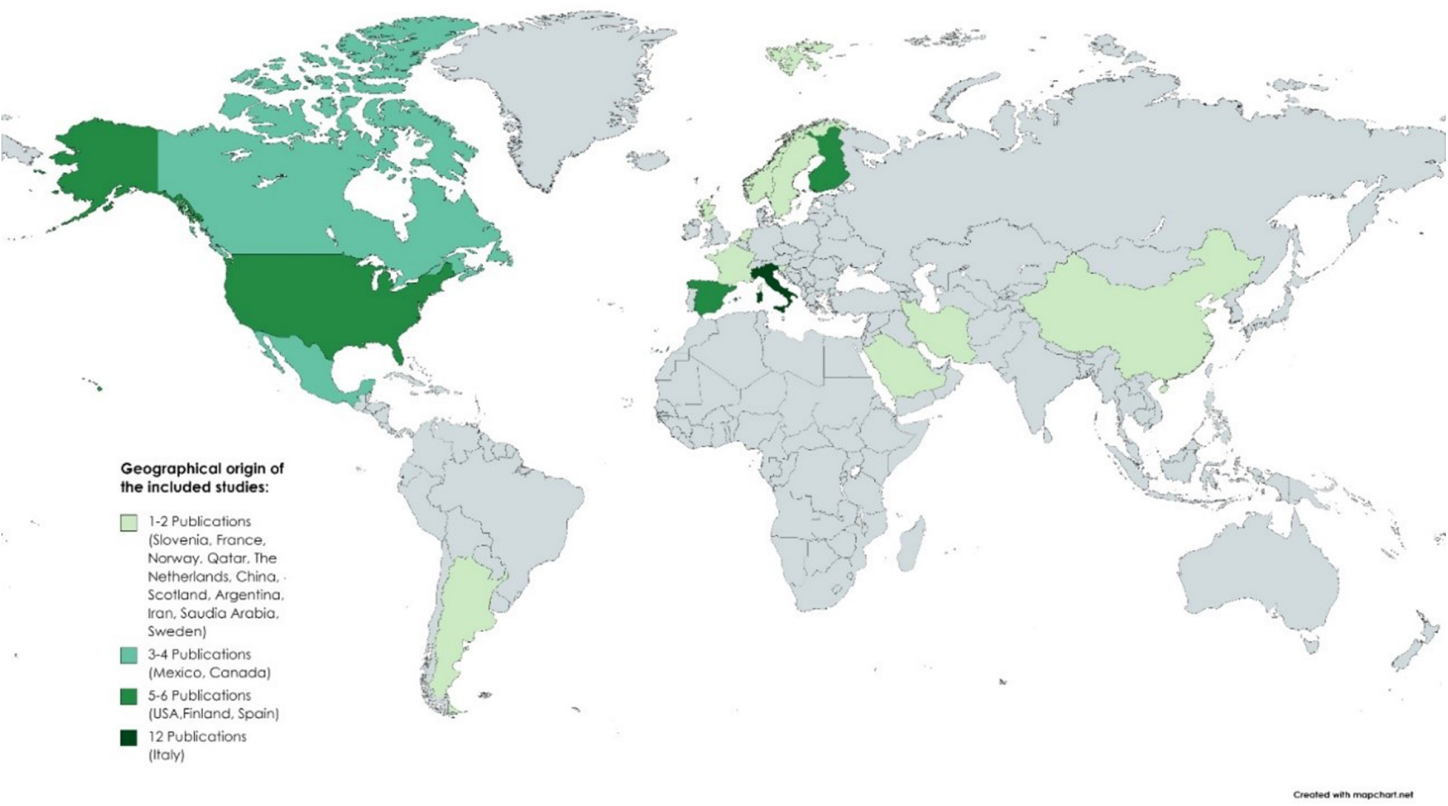
Geographical distribution of the selected studies. No differentiation was made between studies conducted in one country and multicentric studies (conducted in more than one country). The majority of studies were conducted in Europe and North America, with Italy as the country with the highest number of studies.

Most studies were conducted in Europe and North America; few in South America, North Africa, and South Asia; and none in Australia (Figure 2). Considering the single-country studies, the majority were from Italy (n = 8), and multicountry studies were conducted in the USA and Italy (n = 2), Canada and Argentina (n = 1), France and Italy (n = 1), and Italy and Slovenia (n = 1). However, two studies did not mention the geographical origin of the studied population (11, 12). As to the year of publication, most studies were published in recent years, particularly over the last 5 years, in 2020, 2021, and 2023 (n = 7 each), 2018 and 2022 (n = 5 each), and 2019 (n = 4), followed by 2016 and 2013 (n = 3 each) and 2017, 2012, and 2009 (n = 2 each), with 2008 (n = 1) as the year of the oldest publication (8).

When looking at the participants’ selection approach, 7 studies mentioned both the inclusion and exclusion criteria, 3 mentioned only the inclusion criteria, and 13 mentioned only the exclusion criteria, while 25 studies did not mention them. Regarding age range and sample size (including controls), only 1 study focused on newborns (sample size = 127), 4 on newborns during their growth into childhood (sample size from 20 to 85), and 17 on children (sample size from 18 to 1478), with adults as the most studied population in 18 publications (sample size from 18 to 132). Yet, 4 publications compared children and adults (sample size from 19 to 61), and 4 studies did not mention age (sample size from 6 to 80). Regarding gender, although 14 studies did not mention this criterion, all other studies included both sexes, except 1 that only studied female children (22). A parameter rarely discussed in the analyzed publications was ethnicity, with only two publications assessing this criterion (13, 23). Dietary habits were not considered, except in RCT studies.

Since age is a prominent factor in CeD onset and progression, we grouped the selected studies as newborns, children, and adults. Since some studies compared CeD patients of different ages, two other groups were also created, newborns/children and children/adults. We first presented a brief description of CeD patients and test conditions of each publication within each age group (Table 2). Afterward, we analyzed the NGS approaches/methodologies used (Table 3). Finally, we compiled a brief summary of the major gut microbiome characterization findings (Table 4).

**Table 2:** Characterization of reviewed studies regarding participant information (controls and CeD patients), tissue sample, and type of study.

Articles	Control sample	CeD sample	Biopsy diagnosis	CeD GFD	Tissue sample	Family risk factor	Type of study
Newborns							
Olivares et al. (24)	Full-term newborns with at least one first-degree CeD relative (n = 127)		–	No	Feces	HLA DQ 2/8	Prospective
Newborns and children							
Sellito et al.^a^ (10)	–	11 5	–^b^	Yes No	Feces	ND	Prospective
Olivares et al. (25)	10 (matched)	10	No	No	Feces	HLA DQ 2/8	Prospective
Leonard et al. (26)	USA (n = 18) and Italy (n = 13) children with first^-^degree CeD relative from CDGEMM study		–	No	Feces	Yes	Prospective
Leonard et al. (27)	54 (developed CeD autoantibodies) 31 (confirmed CeD)		Yes	Before/after	Feces Blood	Yes	Prospective
Children							
Barbato et al.^a^ (8)	8	10	Yes	Before/after	Duodenum	–	Prospective
Ou et al. (28)	18	33 17 3	Yes	No Yes (>7 m) Yes (>1 m)	Duodenum	–	Cross-sectional
Meij et al. (29)	21 (matched)	21	Yes	No	Feces	–	Cross-sectional
Pozo-Rubio et al. (30)	55 (children with first-degree CeD relative)		–	No	Feces Blood	HLA DQ 2/8	Prospective
Quagliariello et al. (18)	16 (control group)	20 (probiotic group) 20 (placebo group)	Yes	Yes Yes	Feces	–	Prospective
Rahmoune and Boutrid^a^ (15)	648^c^	424^c^	–	–	–	–	Cross-sectional
Rintala et al. (22)	18	9	Yes	No	Feces	Yes	Prospective
Zafeiropoulou et al. (17)	18 (siblings) 57 (healthy)	45 20 (new-onset CeD)	Yes	Yes No	Feces	–	Cross-sectional and Prospective
Sample et al. (31)	17 (siblings)	22	Yes	Before/after	Feces	–	Cross-sectional
Leonard et al. (16)	10 (healthy matches)	10	Yes	–	Feces	–	Cross-sectional and Prospective
Singh et al. (32)	18 (T1DM) 12 (healthy)	19 9 (CeD and T1DM)	Yes	–	Feces Blood	–	Cross-sectional
Biase et al. (33)	16	21	Yes	No	Feces Duodenum	HLA DQ 2/8	Cross-sectional
Mouzan et al. (34)	20 (healthy) 19 (non-CeD)	40	Yes	No	Feces Duodenum	–	Cross-sectional
Milletich et al. (35)	1,452^d^	26^d^	Yes	No	Feces	HLA DQ 2/8	Prospective
Mouzan et al. (36)	39	40	Yes	–	Feces Duodenum	–	Cross-sectional
Aguayo-Patrón et al. (37)	17	18 (genetic risk) 18 (genetic risk + autoantibodies)	ND	– –	Feces Blood	Yes	Cross-sectional
Girdhar et al. (38)	16 (2.5 y.) 13 (5 y.)	15 (2.5 y) 9 (5 y)	Yes	No	Feces Blood	Yes	Prospective
Adults							
Nistal et al. (39)	9	9	Yes	No	Feces	–	Cross-sectional
Garcia-Mazcorro et al. (40)	12 (NCGS) 12 (healthy)	6	Yes	Yes	Feces Duodenum Blood	CeD relatives: 67% HLA DQ 2/8: 83%	Prospective
Bodkhe et al. (41)	15 (CeD patients prior to disease) 24	23	Yes	No	Feces Duodenum	–	Cross-sectional
Serena et al. (42)	10	10 (active) 8 (in remission)	–	No Yes	Feces Blood	–	Cross-sectional
Francavilla et al. (19)	–	54 (probiotic group) 55 (placebo group)	–	Yes Yes	Feces	–	Prospective
Panelli et al. (43)	6 (potential CeD) 31 (non-CeD)	13 (active CD) 29 (TCD) 4 (refractory CD)	Yes	No Yes Yes	Feces Duodenum	–	Cross-sectional
Nylund et al. (44)	10 (NCGS) 14 (healthy)	19	–	Yes	Feces	–	Cross-sectional
Smecuol et al. (20)	–	7 (placebo group) 5 (treatment group)	Yes	Yes Yes	Feces	–	Prospective
Bibbó et al. (45)	–	30 20 (CeD + AD)	Yes	Yes Yes	Feces	HLA DQ 2/8	Prospective
Naseri et al. (46)	15 (healthy) 30 (IBS) 12 (NCGS)	15	Yes	–	Feces	–	Cross-sectional
Nobel et al. (47)	8 (NCGS) 8 (healthy)	9	Yes	Yes	Feces	–	Prospective
Schiepatti et al. (48)	4 (PCD)	8 18 (no symptoms) 3 (diarrhea) 4 (other symptoms)	Yes	No Yes Yes Yes	Feces Duodenum	Yes	Prospective
Andriulli et al. (21)	–	13 (placebo group) 19 (bread, 3 g GF) 18 (bread, 6 g GF)	Yes	No Yes Yes	Feces Blood Urine	HLA-DQ2/8	Prospective
Constante et al. (49)	30	11	24	Yes	Feces Duodenum	–	Cross-sectional
Shi et al. (23)	30	30	Yes	–	Feces	–	Cross-sectional
Francavilla et al. (50)	66	63 3	Yes	Yes No	Feces	Yes	Cross-sectional
Naseri et al. (51)	15 30 (IBS) 12 (NCWS)	15	Yes	NA	Feces	–	Cross-sectional
Herfindal et al. (52)	–	39 (LFD and GFD) 36 (GFD)	–	Yes Yes	Feces	–	Prospective
Children and adults							
Kalliomäki et al. (53)	9 (control children)	10 children 6 adults	Yes	No Yes	Duodenum Duodenum	–	Cross-sectional
Nistal et al. (54)	5 (children) 5 (adults)	8 children 5 adults 5 adults	–	No Yes No	Duodenum Duodenum Duodenum	–	Cross-sectional
Cheng et al. (55)	28 (children) 23 (adults) 10 (children)	10 children 6 adults	Yes	No Yes	Feces Duodenum	–	Cross-sectional
Cheng^a^ (13)	9 (children)	10 children 6 adults	Yes	No Yes	Feces Duodenum	–	Prospective
Age not mentioned							
Ciccocioppo et al.^a^ (11)	–	–	–	–	Feces Duodenum Saliva	–	–
Gutierrez et al.^a^ (9)	12 (NCGS) 12 (controls)^e^	6	–	Before/after	Feces Duodenum	–	Prospective
Faroug^a^ (12)	23 (controls)	30 (ACD) 24 26 (RCD)	–	No Yes Yes	Duodenum	–	Cross-sectional
Nylund et al.^a^ (14)	10 (NCGS) 14 (controls)	19	–	–	Feces	–	Cross-sectional

m, month; y, years; AD, autoimmune disease; NCGS, non-celiac gluten sensitivity; T1DM, type 1 diabetes mellitus; IBS, irritable bowel syndrome; ACD, active celiac disease; NCWS, non-celiac wheat sensitivity; PCD, potential celiac disease (on a gluten-containing diet); RCD, patients with refractory to a GFD.

^a^ Gray literature.

^b^ Only in relatives.

^c^ Brief report from four European tertiary care hospitals, in Paris (France) and Milan, Udine, and Perugia (Italy).

^d^ From the ABIS prospective investigation, a population-based cohort of 17,055 children.

^e^ Negative celiac antibodies, with persistent IBS-type symptoms, on a strict GFD.

**Table 3:** Microbiome analysis workflow of each publication: pre-analysis (DNA extraction and library preparation steps), NGS technology and target rDNA 16S region, and microbiome analysis approach presented to highlight the diversity of methodological approaches used in the reviewed publications.

Articles	Pre-NGS		NGS		Analysis			
	DNA extraction	Library preparation	technology mentioned	16S region	Alpha diversity	Beta diversity	OTU composition	Relative abundance
Newborns								
Olivares et al. (24)	–	–	16S rRNA seq	–	–	–	–	–
Newborns and children								
Sellito et al.^a^ (10)	–	–	16S rRNA seq	–	–	–	–	–
Olivares et al. (25)	FastDNA Spin kit for soil	–	Illumina	V1–V2	X	X	X	X
Leonard et al. (26)	PowerSoil DNA extraction kit	–	16S rRNA seq	–	–	X	–	–
Leonard et al. (27)	–	–	–	–	–	–	–	–
Children								
Barbato et al.^a^ (8)	–	–	16S rRNA seq	–	–	–	–	–
Ou et al. (28)	Phenol–chloroform	–	16S rRNA seq	–	–	–	–	–
Meij et al. (29)	QIAamp DNA Mini kit	–	16S rRNA seq	–	–	–	–	X
Pozo-Rubio et al. (30)	QIAamp DNA Stool Mini kit	–	qPCR	–	–	–	–	X
Quagliariello et al. (18)	QIAamp DNA Stool Mini kit	Nextera XT Library Prep kit	Illumina	V3–V4	X	–	–	–
Rahmoune and Boutrid^a^ (15)	–	–	–	–	–	–	–	–
Rintala et al. (22)	GXT Stool Extraction kit	PCR with custom-designed dual-indexed primers	16S rRNA seq	–	X	–	X	X
Zafeiropoulou et al. (17)	Chaotropic	–	Illumina	V4	X	X	X	–
Sample et al. (31)	DNeasy PowerSoil DNA kit	MS Library Prep protocol	Illumina	V3–V4	X	X	X	–
Leonard et al. (16)	PowerSoil DNA extraction kit	Nextera XT Library Prep kit	Illumina	–	–	–	–	–
Singh et al. (32)	QIAamp Fast DNA Stool Mini kit	–	Illumina	V3–V4	X	X	–	–
Biase et al. (33)	DNeasy Blood and Tissue Mini kit	–	Bacterial 16S rRNA HTF-Microbi.Array	–	–	–	–	–
Mouzan et al. (34)	DNeasy PowerSoil DNA kit	Nextera XT Library Prep kit	Shotgun metagenomic analysis	–	X	X	–	–
Milletich et al. (35)	–	–	Illumina	V3–V4		X	X	–
Mouzan et al. (36)	–	–	HiSeq platform	–	–	–	–	–
Aguayo-Patrón et al. (37)	QIAamp Fast DNA Stool Mini kit	–	Illumina	V3–V4	X	X	X	X
Girdhar et al. (38)	MagAttract Microbial kit	NGS Library Quantification Complete kit	Illumina	V4	X	X	–	–
Adults								
Nistal et al. (39)	–	–	16S rRNA seq	V4	X	X	X	–
Garcia-Mazcorro et al. (40)	DNA extraction kit	TruSeq DNA Library Prep	Illumina	V4	X	X	X	–
Bodkhe et al. (41)	QIAamp Fast DNA Stool Mini kit	–	Illumina	V4	–	–	X	–
Serena et al. (42)	QIAamp Blood Midi + DNeasy PowerSoil extraction kits	–	Illumina	V4	X	X	–	X
Francavilla et al. (19)	FastDNA Spin kit for soil	NEBNext multiplex small RNA Library Prep set	16S rRNA seq	V3	X	X	–	–
Panelli et al. (43)	QIAamp DNA Stool Mini kit + DNeasy Blood and Tissue kit	Nextera XT Library Prep kit	Illumina	V3–V4	X	X	X	–
Nylund et al. (44)	–	–	Illumina	V3–V4	X	X	X	X
Smecuol et al. (20)	–	–	Illumina	V3	X	X	–	X
Bibbó et al. (45)	QIAamp Fast DNA Stool Mini kit	MS Library Prep protocol	Illumina	V4	X	–	X	X
Naseri et al. (46)	–	–	16S rRNA seq	–	–	–	–	X
Nobel et al. (47)	MagAttract PowerSoil kit	Nextera DNA Flex Library Prep kit	Illumina	V3–V4	X	X	–	X
Schiepatti et al. (48)	DNeasy Blood and Tissue kit	Nextera XT Library Prep kit	Illumina	V3–V4	–	–	X	–
Andriulli et al. (21)	DNeasy Blood and Tissue kit + DNA PowerFecal kit	MS Library Prep protocol	Illumina	V3–V4	X	X	–	X
Constante et al. (49)	–	–	Illumina	V3–V4	–	X	–	–
Shi et al. (23)	FastDNA Spin kit for soil	–	Illumina	V3–V4	X	X	–	X
Francavilla et al. (50)	DNeasy PowerSoil Pro Kit	–	Illumina	–	X	–	–	–
Naseri et al. (51)	QIAamp DNA Stool Mini kit	–	qPCR	–	–	–	–	X
Herfindal et al. (52)	Mag Midi LGC kit	–	16S rRNA seq	–	X	X	X	X
Children and adults								
Kalliomäki et al. (53)	–	–	16S rRNA seq	–	–	–	X	–
Nistal et al. (54)	DNeasy Blood and Tissue kit + NucleoSpin Tissue XS kit	–	16S rRNA seq	V1–V9	–	–	X	–
Cheng et al. (55)	–	–	16S rRNA seq	–	–	–	X	–
Cheng^a^ (13)	Bead-beating with phenol–chloroform	–	16S rRNA seq	–	–	–	–	–
Age not mentioned								
Ciccocioppo et at.^a^ (11)	–	–	–	–	–	–	–	–
Gutierrez et al.^a^ (9)	–	–	16S rRNA seq	–	–	–	X	–
Faroug^a^ (12)	–	–	16S rRNA seq	–	–	–	–	–
Nylund et al.^a^ (14)	Bead-beating with KingFisher	–	16S MiSeq	–	–	–	–	–

Although some pre-analysis consumables are similar between some studies, their combination is unique, with no publication reproducing the same DNA extraction and library preparation approach, even when considering publications from the same authors. The same diversity is observed when looking at the target rDNA 16S region of interest and the microbiome analysis approach. Other pre-NGS steps and information on the software used for microbiome analysis were deliberately omitted to improve clarity in table interpretation. The presented information is enough to demonstrate the unicity of each publication microbiome analysis workflow, obtained by the cumulative combination of different approaches in each step of the workflow.

^a^ Gray literature.

**Table 4:** Microbiome composition described in reviewed publications, regarding the former phyla *Firmicutes*, *Actinobacteria*, *Bacteroidetes*, *Proteobacteria*, *Fusobacteria*, and *Verrucomicrobia* and genera *Bacteroides* and *Lactobacillus*.

Articles	Firmicutes	Bacteroidetes	Fusobacteria	Proteobacteria	Verrucomicrobia	Actinobacteria	*Bacteroides*	*Lactobacillus*	Other groups
Newborns									
Olivares et al. (24)	–	–	–	–	–	–	–	–	S
Newborns and children									
Sellito et al.^a^ (10)	HA overall	HA controls	–	–	–	HA overall	Present	HA overall	S, G, P
Olivares et al. (25)	Present^b^	Present	–	Present	–	Present	–	–	S, G, P
Leonard et al. (26)	Present	Present	–	–	–	–	–	–	S, G
Leonard et al. (27)	–	–	–	–	–	–	–	–	–
Children									
Barbato et al.^a^ (8)	–	–	–	–	–	–	–	–	–
Ou et al. (28)	Present	Present	Present	LA	–	HA overall	–	–	S, G ,P
Meij et al. (29)	Present	Present	–	–	–	Present	HA	HA overall	S, G, P
Pozo-Rubio et al. (30)	–	–	–	–	–	–	Present	Present	S, G
Quagliariello et al. (18)	Present	LA^b^	–	Present	Present	LA^b^	Present	Present	S, G, P
Rahmoune and Boutrid ^a^ (15)	–	–	–	–	–	–	–	–	–
Rintala et al. (22)	–	–	–	–	–	–	Present	Present	G, P
Zafeiropoulou et al. (17)	Present	–	–	–	–	LA	Present^b^	Present^b^	S, G, P
Sample et al. (31)	–	–	–	–	–	–	Present^b^	Present	S, G
Leonard et al. (16)	–	–	–	–	–	–	LA^b^	–	S, G
Singh et al. (32)	Present	HA	–	HA^b^	Present	HA overall	–	–	G, P
Biase et al. (33)	–	HA^b^	–	HA	–	–	LA^b^	–	G, P
Mouzan et al. (34)	HA overall^b^	HA overall^b^	–	HA overall	HA overall	LA	LA	HA^b^	S, G, P
Milletich et al. (35)	–	–	–	–	–	–	Present	–	G
Mouzan et al. (36)	–	–	–	LA	–	–	LA	–	S
Aguayo-Patrón et al. (37)	Present	Present	–	HA overall	Present	Present	Present	–	G, P
Girdhar et al. (38)	–	–	–	–	–	–	Present	–	S, G, P
Adults									
Nistal et al. (39)	HA overall	–	Present	HA overall	–	HA overall	–	HA overall	G, P
Garcia-Mazcorro et al. (40)	HA overall	LA overall^b^	LA^b^	Present	–	HA overall	–	–	G, P
Bodkhe et al. (41)	Present^b^	Present^b^	–	Present	–	Present	Present	Present	S, G, P
Serena et al. (42)	Present	HA overall	–	HA overall	–	HA overall	Present	–	G, P
Francavilla et al. (19)	–	–	–	–	–	Present	–	Present	S, G, P
Panelli et al. (43)	LA^b^	Present^b^	Present^b^	HA^b^	–	LA	HA	–	S, G, P
Nylund et al. (44)	–	–	–	–	–	Present	–	–	S, G, P
Smecuol et al. (20)	–	–	–	–	–	–	–	–	S, G
Bibbó et al. (45)	Present^b^	Present	–	Present	Present	Present^b^	LA^b^	–	G, P
Naseri et al. (46)	HA^b^	Present	–	–	–	Present	–	LA overall	G, P
Nobel et al. (47)	LA	HA	–	HA	–	–	–	–	P
Schiepatti et al. (48)	LA^b^	–	–	Present	–	Present	–	–	G, P
Andriulli et al. (21)	–	–	–	Present	–	–	–	Present	S, G
Constante et al. (49)	HA overall^b^	Present^b^	–	–	–	–	Present^b^	Present	S, G, P
Shi et al. (23)	Present^b^	HA overall^b^	–	HA^b^	–	HA overall	–	HA^b^	G, P
Francavilla et al. (50)	LA	Present	Present	–	Present	Present	–	–	S, G, P
Naseri et al. (51)	HA^b^	Present^b^	–	–	–	–	–	LA^b^	G, P
Herfindal et al. (52)	HA overall^b^	HA overall	Present	HA overall	–	Present	Present	–	G, P
Children and adults									
Kalliomäki et al. (53)	Present	–	–	–	–	Present	Present	–	S, G
Nistal et al. (54)	Present	–	HA overall	HA overall	–	HA overall	–	–	S, G, P
Cheng et al. (55)	–	HA overall	–	HA overall	–	Present	Present	–	S, G, P
Cheng^a^ (13)	HA overall	Present	–	HA	–	HA overall	Present	–	S, G, P
Age not mentioned									
Ciccocioppo et at.^a^ (11)	–	–	–	–	–	–	–	–	–
Gutierrez et al.^a^ (9)	–	–	–	Present	–	–	–	–	S
Faroug^a^ (12)	–	–	–	–	–	Present	–	–	S, G, P
Nylund et al.^a^ (14)	–	–	–	–	–	–	–	–	P

Results with statistical significance are highlighted, as well as information if members from other taxonomy groups are mentioned in the analyzed publications. “Present” indicates that the phyla or genera were mentioned but with no information on alpha and beta diversity to allow a comparative interpretation of results.

HA, high abundance; LA, low abundance; S, species; G, genus; P, phylum.

^a^ Gray literature.

^b^ With statistical significance.

### 3.2 Characterization of CeD patients, controls, and NGS tissue samples

Newborns were the exclusive study sample of 1 prospective study, with a sample size of 127 participants (24). Since the population regarded newborns, the study focused on undiagnosed CeD (UCeD) patients and did not clarify how the participants were diagnosed later in life. Only the presence of HLA DQ 2/8 genes in family members of the surveyed subjects was mentioned. Feces were the tissue samples tested for microbiome analysis.

In 4 prospective studies that assessed both newborns and children, 2 studied UCeD participants, while the other 2 studied UCeD and treated (TCeD) participants, i.e., under a GFD. One study focused on gluten introduction to diet at 6 months of age (25), comparing 10 CeD patients to 10 best-matched controls, all with HLA-DQ2/8 genes, selected from a larger case–control study, namely, PROFICEL, with samples from 4 to 6 months of age until early childhood (in some cases after 5 years). Another study included samples available at birth, at 3 and 4–6 months old, and at 12 months old, from participants from the CDGEMM cohort (26). Two studies only mentioned that the infants had a familial risk for the disease but did not mention if HLA DQ 2/8 genes were tested. Duodenum biopsy gold standard diagnosis was performed in 1 study (Leonard et al., 2023) and only in the relatives of the enrolled CeD patients in another (27). Feces were tested in all studies and blood in 1 study.

From the 17 studies focused on CeD children, 5 studied UCeD patients, 3 studied TCeD, and 2 studied both UCeD and TCeD, while 7 did not mention this parameter. Regarding the studies on TCeD patients, the age at which GFD started and its duration varied between studies and participants. Only 6 articles mentioned the genetic factor in the studied subjects and family members, varying from 8% to 100%. Regarding duodenum biopsy diagnosis, 14 studies mentioned to have performed it, while the other 3 did not. Feces were tested in 14 studies, duodenum biopsies in 5, blood in 4, and 1 study did not mention the used tissue samples.

Of the 18 publications regarding CeD in adults, 9 investigated TCeD patients, 3 studied UCeD, and 3 studied both UCeD and TCeD, while 3 did not mention this parameter. TCeD patients were all in a GFD, but in 1 study, a 14-day gluten challenge was conducted (47). Family genetic history was only mentioned in four publications. Duodenum biopsy was mentioned in 13 publications. All studies mentioned the used sample, with feces being tested in all studies, 5 tested duodenum biopsies, 3 tested blood, and 1 tested urine.

CeD children and adults were compared in 4 publications, both UCeD and TCeD patients. Family history was mentioned in none, but the gold standard diagnosis was performed in 3 of the 4 studies. All tested duodenum biopsies, and 2 also tested feces.

Finally, 4 publications did not mention age. Of those, only 2 indicated the selected population, both UCeD and TCeD patients. Family history and the gold standard diagnosis were not indicated. Feces and duodenum biopsies were analyzed in 3 studies and saliva in 1 study.

Subjects selected for the role of controls in the selected publications varied according to the aim of the study, ranging from healthy controls (20 publications), age- and/or sex-matched controls (6 publications), non-CeD (5 publications), placebo controls (4 publications), healthy and non-CeD siblings (2 publications), TCeD (2 publications), controls with other diseases (1 publication), and non-CeD siblings (1 publication). However, 7 studies did not mention this parameter, 5 involving adults, 1 involving newborns and children, and 1 without age information.

### 3.3 Methodologies used for NGS microbiome analysis

A microbiome analysis comprises the following sequential steps: DNA extraction, library preparation, DNA sequencing, data processing and quality control (quality check, trimming, denoising, alignment, and phylogeny), sequence analysis/microbiome characterization (reference-based and diversity-based), and statistical analysis with the independent variables under study (56). In the reviewed manuscripts, several did not include all these steps, but some studies did, some of which in great detail. The most significant aspect of this analysis was the variability of techniques used in the different manuscripts under revision, at all steps. In addition, not many papers fully described their sequencing, analysis, and interpretation workflow. Table 3 summarizes the retrieved information from the 48 publications under review regarding NGS sequencing and microbiome analysis. Briefly, DNA extraction information was available only in 33 studies, with all except 4 mentioning to have used commercially available kits. Of note was the huge diversity of the used kits, almost equal to the number of studies that mentioned them. Regarding DNA library preparation, only 15 studies mentioned it, as well as the used kits (except 2 studies), mainly from Illumina®. As to DNA sequencing, the most commonly used technology was Illumina®, mentioned in 26 publications, while 2 mentioned to have used 454 pyrosequencing (Roche®). Others mentioned qPCR 16S rRNA (n = 3), metagenomic sequencing (n = 1), shotgun metagenomic (n = 1), flow cytometry analysis combined with qPCR (n = 1), and bacterial 16S rRNA HTF-Microbi.Array (n = 1). Of note, qPCR and microarrays are not sequencing techniques. The most frequently tested 16S rDNA targeted regions were V3–V4 (n = 12), followed by V4 (n = 7), V3 (n = 2), V1–V2 (n = 1), V1–V6 (n = 1), and V1–V9 regions (n = 1). Nonetheless, 25 publications did not mention the DNA region analyzed.

Sequencing data processing was not mentioned in 20 studies. Those that did indicated the use of a single software or a combination of software. The Quantitative Insights into Microbial Ecology (QIIME™) software (n = 8) was the most frequent. Quality control, such as sequence quality examination and trimming, was indicated only in 15 publications, and denoising was mentioned only in 12 publications, all using Divisive Amplicon Denoising Algorithm (DADA) 2. Sequence alignment methodologies were indicated only in 7 publications. Microbiome statistical analysis was frequently mentioned, mainly performed using R software packages (n = 12), with 23 studies describing the conducted OTU analysis and 13 studies specifying the statistical tests applied.

### 3.4 Sequence analysis/microbiome characterization

Two central approaches are used in microbiome characterization: analyzing the taxa composition of the sample (at various taxonomic levels, typically from phylum to species) and interpreting the ecological diversity, or richness, of the microbial community under analysis (typically considering its alpha and beta diversity, i.e., the diversity inside each community/sample and the comparative diversity between communities/samples, respectively).

Regarding community diversity, the preferred approach to determine alpha diversity was Shannon and Chao1 indexes, mentioned in 9 and 5 publications, respectively, with both being used in 4 publications and 2 publications indicating to have assessed this parameter but with no reference to the method (17, 38). To study gut microbiome composition regarding bacterial communities, OTU composition comparisons were the most used approach, through the application of one or more different statistical techniques. Yet, 31 publications did not share details about this parameter. Regarding relative abundance comparison, it was assessed with various methods in 12 studies. However, 36 publications did not share details on this parameter (Table 3).

Finally, 9 publications detailed the methods used to study microbiome results against other variables under study, including Spearman coefficient (n = 2), Kruskal–Wallis test (n = 2), MaAsLin (n = 1), Wald parametric test (n = 1), DESeq2 (n = 1), Wilcoxon signed-rank test or the Mann–Whitney test, ANOSIM and Adonis tests (n = 1), PICRUSt (n = 1), and Friedman test (n = 1). The remaining publications did not use this analysis approach in their studies.

#### 3.4.1 Gut microbiome diversity/richness

Microbial richness, or diversity, can be defined by the total number of different taxa present in the microbial community under study. However, the proportion of each taxa representative within the overall number of detected reads (or microorganisms) is also important to better interpret the microbial ecosystem. Some mathematical indexes are available, with Chao1 and Shannon diversity indexes being the most frequently used (56). Chao1 is an indicator of species richness (total number of species in a sample) that is sensitive to rare OTUs (singletons and doubletons), while the Shannon diversity index evidences how evenly the different taxa are distributed in the sample. When combined, they provide an overview of how balanced, or diverse, the studied microbiome is. A high richness score means the studied microbiome is balanced with different taxa, which is typically linked to good health, while lower richness scores suggest a less diverse microbiome, with fewer types of microorganisms, which is linked to lower health (56, 57).

Microbial richness was not investigated in the study on newborns, yet it was assessed in 1 of the 4 prospective studies regarding newborns and children, with the healthy controls showing a statistically relevant increased richness and diversity during the study period in contrast to the children that developed CeD, with no increase in their gut microbial diversity (25). From the 17 studies focusing on children, only 1 observed statistically relevant differences in microbial composition between CeD children and the controls (31), while 2 observed no statistically relevant changes in microbiome richness in CeD patients compared to the controls (16, 31). Regarding adults, 1 study observed that the fecal bacterial richness was higher in non-CeD controls but without reaching statistical relevance (39), 2 studies found no notable difference between study groups (43, 44), and 1 study observed no important differences when comparing gut microbiome of patients and controls (40). Moreover, 1 study observed that microbiome richness was correlated with the duration of CeD, but no remarkable differences between groups were obtained (45). Another study found relevant differences in microbial community richness between CeD and control groups (23). Lastly, 1 RCT that studied the impact of a GFD FODMAP diet (a diet low in fermentable oligo-, di-, and monosaccharides and polyols) against regular GFD and observed that after the 4 weeks, FODMAP diet led to changes in the overall community structure of the fecal microbiota, whereas low FODMAP diet had no impact on fecal bacterial richness or evenness (52). However, 11 publications did not mention this parameter. Of the 4 studies comparing children and adults, only 1 mentioned that bacterial richness was remarkably lower in children, whether they were healthy or CeD patients (54). Of the 4 publications with no age information, 1 showed that neither diet (GFD vs. regular diet) nor disease status impacted gut microbiome richness or diversity (9), and another showed no differences in richness between study groups.

#### 3.4.2 Gut microbiome composition

Microbial taxonomy is constantly and rapidly evolving, due to 2 main aspects: the rapid evolution of available technology used to classify microbial entities and the constantly increasing number of new species being discovered and, thus, classified. Thus, it is not infrequent not only the rise of novel taxa, particularly at the species and genus level, but also the update of taxa nomenclature. From time to time, these changes go up on the taxonomy hierarchy, changing also the nomenclature used at higher levels of the universal phylogeny rank (species, genus, family, order, class, phylum, clade, domain). In this review, we used the nomenclature present in the analyzed publications. For future annotation purposes, we included the new nomenclature in the subtitle of each taxon analyzed.

**Figure 3:**
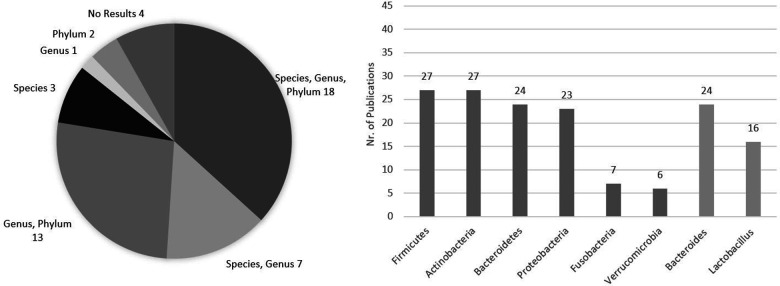
Frequency of taxonomy levels mentioned in the analyzed publications and number of publications specifically addressing the former phyla Firmicutes, Actinobacteria, Bacteroidetes, Proteobacteria, Fusobacteria, and Verrucomicrobia and the genera *Bacteroides* and *Lactobacillus* in their microbiome analysis presentation. Total publications = 48.

In regard to gut microbiome composition in the reviewed publications, former phyla Firmicutes, Actinobacteria, Bacteroidetes, Proteobacteria, Fusobacteria, and Verrucomicrobia and genera Bacteroides and Lactobacillus were the taxonomy levels more frequently mentioned and discussed in the reviewed publications. Firmicutes and Actinobacteria were mentioned in 27 studies, Bacteroidetes in 24, Proteobacteria in 23, Fusobacteria in 7, and Verrucomicrobia in 6. Regarding genera, *Bacteroides* was found in 24 studies and *Lactobacillus* in 16 (Figure 3). In more detail, 18 publications investigated their microbiome data up to the species level, also mentioning the genus and phylum, while 13 publications analyzed their results only at the genus and phylum levels, 7 at species and genus levels, 3 at the species level, 2 at the phylum, and 1 at the genus level. Lastly, 4 gray literature publications had no microbiome results, 1 abstract, 1 briefing, 1 cohort profile, and 1 poster abstract (11, 15, 18, 27) (Figure 3), mentioning only some technical aspects of the analysis. Additionally, it is relevant to highlight that 3 publications, in parallel (not prior) to the NGS microbiome analysis, also performed a culture-based analysis in their study, using quantitative cultivation methods (28), pure cultures of reference strains (30), and enumeration of fecal cultivable bacteria and microbiome (19). Being out of the scope of this review, these results are not discussed here.

An overall view of the gut microbiome composition findings regarding the most frequently mentioned and discussed phyla (Firmicutes, Actinobacteria, Bacteroidetes, Proteobacteria, Fusobacteria, Verrucomicrobia) and genera (*Bacteroides* and *Lactobacillus*) in the studies under revision is described in Table 4. Of note are the contradictory results between studies regarding each of these phylogenetic groups, as discussed below.

##### 3.4.2.1 *Firmicutes* (currently Bacillota)

The study on newborns did not mention Firmicutes (24). In the studies on newborns and infants, 1 observed that the subjects who harbored a notable higher proportion of Firmicutes at 4 months of age later developed CeD and that healthy infants had an increase in Firmicutes diversity over time, contrarily to those who developed CeD (25). Another study found this phylum as the most predominant, but not equally distributed, in the tested subjects, TCeD and UCeD (10), and 1 study found that 4 to 6 months after birth, infants with high or standard genetic risk (two copies of HLA-DQ2 and heterozygous for DQ2 or DQ8, respectively) had a decrease in *Clostridium perfringens* (now *Clostridioides perfringens*) (26).

In the investigations with children, one study that used scanning electron microscopy (SEM) to characterize CeD biopsies indicated that, among other phylogenetic groups, Firmicutes, although present in all samples, were increased in number in those that showed rod-shaped bacteria in SEM (28). Other studies found similar microbial patterns in UCeD children and controls (29); reduction of Firmicutes/Bacteroidetes ratio in CeD subjects (18); increased *Clostridium*, *Ruminococcus*, and *Oscillospira* and decreased *Dialister* in CeD patients (32); *Firmicutes* and *Bacteroidetes* as the most abundant phyla, both in CeD patients and non-CeD controls (34); and no noteworthy difference in Firmicutes abundance between studied groups (Group 1, children with high-risk HLA, negative for autoantibodies; Group 2, children with high-risk HLA, positive for autoantibodies; Group 3, children without a genetic risk for CeD or type 1 diabetes) (37). Finally, 1 article assessed in great detail this phylum up to the species level (17). However, 10 publications did not mention this phylum in their microbiome analysis.

Only 4 studies that focused on adults did not mention this phylum in their results. Of the other 14 studies, 1 indicated a high Firmicutes abundance regardless of disease status (40), 4 found an overall high Firmicutes abundance (23, 39, 49, 52), and the others did not find important differences in Firmicutes abundance between pre-disease and CeD subjects (41) or any correlation between this phylum and disease duration (45). Yet, 2 articles found a decrease in Firmicutes abundance in the CeD group (43, 47), while 1 study indicated this phylum as the most abundant in all samples, but with Firmicutes/Bacteroidetes ratio being statistically significantly higher in CeD subjects compared to that in the other studied groups (46). The same authors, in a different study, found similar results, mentioning a higher Firmicutes abundance and Firmicutes/Bacteroidetes ratio in CeD patients compared to those in the controls (51). On the other hand, one study indicated a decrease in Clostridiales and an increase in Bacillales in CeD patients (42). One study that compared non-anemic patients with CeD patients with iron deficiency anemia observed that the CeD group had a higher relative Firmicutes abundance (48), and another indicated that Firmicutes bacteria such as CAG 83 and *Ruminococcus bicirculans* were reduced in patients with positive anti-TG2 IgA serology (50).

Of the 4 comparative studies on children and adults, only 1 mentioned that *Streptococcus* was present in lower numbers throughout the groups (53), another mentioned a higher rate of *Streptococcus* in controls compared to CeD patients (54), 1 observed overall high Firmicutes abundance (13), and lastly another did not mention this phylum in their results (55).

##### 3.4.2.2 *Bacteroidetes* (currently Bacteroidota)

The study on newborns did not mention Bacteroidetes (24). In the studies on newborns and infants, 1 mentioned Bacteroidetes had no important differences between groups (25), another indicated Bacteroidetes to be colonized and established in the GI tract of non-CeD children during their first year of life (10), and 1 found that 4 to 6 months after birth, infants with high or standard genetic risk (two copies of HLA-DQ2 and heterozygous for DQ2 or DQ8, respectively) had a decrease in *Parabacteroides* (26).

In the investigations with children, the study that used SEM to characterize CeD biopsies also observed Bacteroidetes in all samples and increased in those with rod-shaped bacteria (28). Other studies found similar microbial patterns of Bacteroidetes in UCeD children and controls (29), reduction of Firmicutes/Bacteroidetes ratio in CeD subjects (18), increased *Prevotella* and *Alisipes* in CeD patients (32), CeD patients with a subdominance of Bacteroidetes/*Streptococcus* (33), Bacteroidetes and Firmicutes as the most abundant phyla in both CeD patients and non-CeD controls (34), and no crucial differences in Bacteroidetes abundance between groups (37). However, 10 studies did not mention this phylum.

In the studies on adults, 1 indicated low Bacteroidetes abundance regardless of the disease status (40), another found a marginally lower abundance in the pre-disease group compared to the CeD group (41), while others observed a positive correlation between this phylum and disease duration (45). In 3 other studies, this phylum was overall abundant (23, 42, 52), but others mentioned mixed patterns, with Bacteroidetes being mainly increased in refractory CeD (43), lower in CeD subjects compared to controls (46), or having an increase in relative abundance in CeD patients (47). Another study found that TCeD patients with negative anti-TG2 serology had increased Bacteroidetes abundance (50), while others mentioned that Firmicutes/Bacteroidetes ratio alteration was statistically relevant in CeD patients compared to controls (51) or provided no further information (49). Six studies did not mention this phylum in their results.

Of the 4 studies on children and adults, 1 observed that Bacteroidetes were the major bacterial group in the duodenal mucosa of both CeD patients and controls, with no important differences between the two groups (55), and another also found this phylum as the most predominant in both age groups but with a notable higher abundance in adults compared to children (13).

##### 3.4.2.3 *Fusobacteria* (currently Fusobacteriota)

The study on newborns and the studies on newborns and children did not mention Fusobacteria. In the investigations with children, the SEM study observed Fusobacteria to be present in all samples, being increased in those with rod-shaped bacteria (28). The remaining 16 studies did not mention this phylum. Of the four studies on children and adults, only 1 mentioned this phylum as being overall abundant (54).

Among the 18 studies on adults, only 5 found Fusobacteria, 1 indicating lower abundance in CeD patients (40), another showing that TCeD with positive anti-TG2 IgA serology, which was a symptomatic group without a strict GFD or a short-time of GFD adherence, had a specific reduction of Fusobacteria abundance compared to controls, but between the TCeD groups, no relevant difference was observed (50). Another article found Fusobacteria reduction in potential CeD subjects and an increase of Fusobacteriaceae in mucosal samples of CeD patients (43). Lastly, 2 publications only mentioned having observed this phylum, without further information (39, 52).

##### 3.4.2.4 *Proteobacteria* (currently Pseudomonadota)

No evidence of Proteobacteria was found in the article regarding newborns. Of the studies on newborns and infants, only one mentioned this phylum, but with no relevant results (25). In the children studies, a decrease in CeD patients (28), higher representation in the placebo group (18), notable higher abundance of *Shigella* and *Escherichia coli* in CeD subjects (32), dominance of Enterobacteriaceae in CeD patients (33), overall Proteobacteria abundance in duodenal samples (34), overall increased abundance (37), and Burkholderiales deficiency in CeD patients (36) are described. However, 10 studies did not mention this phylum.

In the studies on adults, 1 identified Proteobacteria as the most abundant in patients on a GFD (40) and *Sutterella* as the lowest after GFD introduction (21), 3 found an increase in Proteobacteria in active CeD subjects (23, 43, 47), and 3 mentioned Proteobacteria to be overall abundant (39, 42, 52). The comparative study between CeD patients with iron deficiency anemia and non-anemic patients observed the latter to have a higher relative abundance of Proteobacteria (48), and another study found no overall difference between groups (41). One study specifically highlighted the Gammaproteobacteria class within overall Proteobacteria abundance in CeD patients with poly-autoimmunity such as autoimmune thyroiditis (45). However, 7 publications did not mention this phylum.

Of the 4 publications that compared children and adults, 2 observed overall Proteobacteria abundance (54, 55), and another found higher levels of this phylum in CeD children (13). Only 1 study did not mention this phylum in their results (53).

##### 3.4.2.5 *Verrucomicrobia* (currently Verrucomicrobiota)

In the studies on newborns, newborns and children, and children and adults, Verrucomicrobia was not mentioned.

In the studies on children, 1 showed that the placebo group had higher Verrucomicrobia abundance (18). Another showed overall increased abundance in the studied samples (34), a trend of higher abundance in non-risk children (37), and lower *Akkermansia* abundance in CeD children compared to children with type 1 diabetes mellitus (32).

In the studies on adults, 1 observed that subjects with TCeD with negative anti-TG2 IgA serology had an increase in *Verrucomicrobia* abundance (50), while the other only mentioned this phylum, without further information (45).

##### 3.4.2.6 *Actinobacteria* (currently Actinomycetota)

In the article about newborns, Actinobacteria was not mentioned. In the studies on newborns and children, 1 observed that Actinobacteria presence early in life in infants at CeD familial risk was influenced by the HLA-DQ2/8 genotype and possibly by other genetic and environmental factors (25). Another study only mentioned overall Actinobacteria abundance (10).

In studies on children, 1 observed overall Actinobacteria abundance in the proximal small intestine (28), while others indicated this phylum to be overall abundant (32) and no difference between CeD patients and controls (29), Actinobacteria reduction in CeD patients (18), decreased *Bifidobacterium* in CeD patients (17), decreased abundance in CeD patients (34), and a trend of higher abundance in non-CeD risk children (37).

Of the 18 studies focused on adults, only 5 did not mention this phylum, of which 4 mentioned an overall abundance (23, 39, 40, 42). The other studies observed a noteworthy higher abundance of Actinobacteria in pre-disease status compared to CeD, but with no important differences between groups (diagnosis, CeD, first-degree relatives, and controls) (41); higher abundance in the probiotic group compared to the placebo group (19); decreased abundance in the active CeD group (43); marginally higher abundance of *Bifidobacterium* in the control group compared to CeD patients group (44); statistically lower relative abundance of *Bifidobacterium* spp. in CeD patients (46); reduced Actinobacteria and *Rothia* spp. in TCeD patients with persistent diarrhea compared to TCeD patients with other persistent symptoms (48); reduced abundance of Actinobacteria in TCeD patients with negative anti-TG2 IgA serology (50); a tendency to lower abundance of Actinobacteria, mainly *Bifidobacterium*, in the FODMAP diet group compared to controls (52); and lower abundance of Actinobacteria in CeD patients with poly-autoimmunity such as autoimmune thyroiditis (45).

Of the 4 studies on children and adults, 1 found *Bifidobacterium catenulatum* in a small number of biopsies (53), 2 found an overall abundance of Actinobacteria (13, 54), and lastly 1 only mentioned Actinobacteria to be present in their studied samples (55).

##### 3.4.2.7 Bacteroides

In the article about newborns, *Bacteroides* was not mentioned (24). In the studies on newborns and infants, 1 mentioned that *Bacteroides* was absent from the gut microbiome of CeD patients up to 24 months old but was predominant in non-predisposed children (10).

In the studies on children, only 4 did not mention this genus. One study found that antimicrobial intake correlated with higher counts of *Bacteroides fragilis* during the first 4 months of life (30). Other publications described different results, with some highlighting an increase of *Bacteroides* in CeD patients and others noting the opposite. In particular, the other studies observed a higher abundance of *Bacteroides vulgatus* and *E. coli* in CeD subjects compared to controls (29), members of the *B. fragilis* group with higher median values in CeD subjects compared to controls but with no relevant differences when compared to the probiotic treatment groups (18), no differences between groups (22), relevant enrichment of *Bacteroides* in CeD and in post-GFD CeD patients compared to healthy controls (31), and *B. vulgatus* at age 2.5 years and *Bacteroides* sp. at age 5 years in the CeD groups but not in controls (38). In contrast, other studies indicated a decrease in *Bacteroides* abundance in CeD patients (16), lower mean relative abundance of *Bacteroides*/*Prevotella* cluster in CeD patients (33), statistically relevant increase in non-CeD controls and a decrease in CeD patients (34), decrease in CeD subjects (36), *Bacteroides* higher abundance in non-CeD risk children (37), and dominance of *Bacteroides*, among others, in the gut microbiome of all tested groups except in the CeD children group. Lastly, 1 article only mentioned the presence of this genus without further information (35), while another assessed in great detail the species within this genus (17).

Of the 18 studies on adults, 1 indicated *Bacteroides* to be higher abundant in pre-disease status compared to CeD subjects (41). Another study found this genus to be predominant in the stool consortium and increased in all CeD groups, including *Bacteroides eggerthii*, which was increased in TCeD (43). Another study observed a weak association between a low baseline abundance of *Bacteroides* with greater symptom response to a low FODMAP diet (52). One study mentioned an important reduction in *Bacteroides* abundance in CeD patients with poly-autoimmunity compared to controls (45), and lastly, 2 studies only mentioned the presence of this genus without further information (42, 49).

Of the 4 studies on children and adults, 1 found *Bacteroides fragilis* in a small number of biopsies (53), another found no difference between groups (55), and lastly 1 only mentioned *Bacteroides* (13).

##### 3.4.2.8 Lactobacillus

In the studies on newborns, children, and adults, and in 3 of the 4 studies on newborns and children, *Lactobacillus* was not mentioned. Nonetheless, the other study on newborns and children mentioned an overall abundance of *Lactobacillus* with no equal distribution between groups (10).

Of the 17 studies on children, 1 determined an overall abundance of *Lactobacillus*, but also not with an equal distribution (29). One study investigating the influence of antimicrobials on the gut microbiome of CeD children determined higher levels of Lactobacillaceae and Gracilibacteraceae in the probiotic and control groups. In addition, *Lactobacillus* spp. had a higher abundance in the control group (18). A different study showed a reduction in *Lactobacillus* after GFD introduction (31), while another showed increased *Lactobacillus* in CeD patients (34). Lastly, 1 study found no differences in this genus abundance between groups (22), and 2 articles only indicated its presence without further information (17, 30).

In the studies on adults, the major findings were that *Lactobacillus* was overall abundant (39), its abundance varied between different diagnosis groups (41) or greatly varied in CeD subjects (21), and also probiotics promote increased lactic acid bacteria, with *Lactobacillus* included (19). In other studies, *Lactobacillus* was the least abundant genus overall, with a lower abundance in CeD subjects (46, 51). Inversely, 1 study reported increased levels of *Lactobacillus* in CeD patients (23). One study only mentioned its presence without further information (49).

Finally, of the 4 publications with no age information, none mentioned Firmicutes, Bacteroidetes, Fusobacteria, Verrucomicrobia, *Bacteroides*, or *Lactobacillus*. Regarding Proteobacteria, only one study mentioned finding a higher abundance of *Pseudomonas* in CeD patients compared to controls, before and after GFD introduction (9). Regarding *Actinobacteria*, another study mentioned its lower levels in patients refractory to GFD (12).

## 4 Discussion

In this review, we analyzed studies from the last 23 years on the gut microbiome of CeD patients using NGS. We analyzed the methodologies used, the characteristics of the studied population, and the observed correlations of the gut microbiome of CeD patients. Synthesizing this information will allow researchers to focus on unexplored questions and improve methodological gaps to enhance reproducibility and comparability, a necessity transversally identified for a long time across all microbiome studies (56–58). Studies focused on CeD are not different, as observed in this current review. Indeed, our evaluation of the selected literature indicated a wide variability of results, with very low concordance between the reviewed studies.

When studying in detail the results regarding the most frequently mentioned and discussed taxa in the selected publications, no universal correlation between a specific species, genus, or phylum presence or abundance and patients with CeD, regardless of their age, GFD status, or even the tissue used to study the gut microbiome, was observed, as mentioned in the Results section.

When examining the technical approaches, a huge variability was also observed. DNA extraction was the most frequently mentioned technique, with an indication of a multitude of different commercially available kits being used, almost to a level of one different kit per publication. This huge variability of DNA extraction methods between studies can greatly contribute to the observed incomparable results (59). However, the most striking observation in this review was the fact that several publications did not describe their technical approaches in detail, which prevents other researchers to replicate their work. This reinforces the reproducibility crisis that has been increasingly impacting science, in a time where the opposite is being advocated and open-access data sharing is being presented as the future of research (60). Indeed, out of the 48 publications analyzed, only 33 mentioned the DNA extraction method, 15 mentioned the library preparation approach, 23 mentioned the analyzed 16S rRNA region, 28 mentioned the data processing approach, and 15 and 12 mentioned the quality control and denoising run, respectively. A similar observation was made regarding data analysis and presentation, with few studies mentioning the used approach, the majority only focusing on presenting the microbiome composition results, and few statistically analyzing its correlation with the participants’ phenotypes or study conditions, apart from the 4 RCT.

Although critical appraisal is only expected in systematic reviews, according to the PRISMA guidelines (7), some points are highlighted in the analysis conducted in this review. Using participants’ inclusion and exclusion criteria as an example, 7 publications mentioned both, 3 only inclusion and 13 only exclusion criteria, while more than half of the reviewed publications (n = 25) did not mention them. This leads us to the conclusion that microbiome scientists, although running observational studies with medical applications or at least medical implications, may not be familiar with the recommendations for these types of research. The Strengthening the Reporting of Observational Studies in Epidemiology (STROBE) guidelines are one of the most frequently used to guide authors in planning and presenting their work clearly and support readers with adequate information for a proper critical appraisal of their work (61, 62). The goal of this review is to aid in solving the inadequacy of research reporting that prevents the assessment of its strengths and weaknesses and, importantly, its generalizability. Since cohort, case–control, and cross-sectional studies are the most frequent ones, the STROBE guidelines focus on these three types of biomedical studies, providing a checklist of 22 items (18 common to all 3) to guide authors (61). Of note is the fact that the STROBE guidelines were published in 2007 and all reviewed studies were published after 2008, with more than half being published over the last 5 years.

Still focusing on participants’ features, the most frequently studied populations were children (n = 17) and adults (n = 18), with some studies comparing both (n = 4). However, the age range was broad in both groups, from 4 months to 18 years in studies on children and from 18 to 65 years in studies on adults. In addition, a few studies that indicated the gender and ethnicity of the patients, as well as other interpersonal heterogeneity factors of human lifestyle and physiology that may affect gut microbiome, such as alcohol consumption and bowel movement, have already demonstrated to contribute to the low concordance between studies if not properly addressed when building the matches between cases and controls (58). As to the number of CeD participants per study, ranging from 9 to 55 (mean = 33) in studies involving children and 9 to 66 (mean = 34) in studies involving adults, it also prevents interpersonal differences from being reduced, further contributing to the low concordance between studies. Power and sample size calculations are essential to allow the delivery of valid conclusions from any biomedical research (63) and need to be better addressed in future work.

As to the tissue samples used in the reviewed studies, all but six studies used feces, which is the most convenient sample to obtain. Interestingly, one-third of the reviewed studies (n = 17) also tested duodenum samples. CeD pathophysiology is tied to the duodenum, and thus studying duodenum samples, although more difficult to obtain, could deliver a more accurate image of the CeD–gut microbiome interplay that the scientific body of research, including medicine, aims to obtain. Moreover, it is also important to notice that how and where the samples are obtained can also influence results. The duodenum, although only 25 cm long, is very diverse. Its abiotic features, such as pH, hormones, or other metabolites, greatly vary between the different duodenum portions, thus influencing its microbiome. The same variability must be considered regarding duodenum luminal aspirates, during the decision on the type of samples to obtain, as well as on the used technique to obtain them (64), as the aspirator can carry material from one portion to another and thus contaminate samples to be used in microbiome studies. No reviewed study that used duodenum samples delivered information on the specific portions tested or the techniques used to obtain and preserve them before analysis. Lastly, when looking at the type of studies reviewed, around half were prospective and half cross-sectional. Only 4 studies were RCT, mainly studying the effect of probiotics in adult CeD patients, having as main conclusions the need to conduct further studies to confirm the hypothesis, which concurs with this review’s overall conclusion on the reviewed studies’ results lacking evidence of a signature gut microbiome.

After analyzing all studies and according to the information provided by the literature that addresses host and technical confounders of microbiome analysis, the main conclusion of this review was the mandatory need to standardize study designs (58–60). Including clinical study approaches to calculate power and sample size, recruiting and grouping an adequate number of participants and samples are also vital, as well as the presentation of results (61–63). All levels of the study must be carefully considered, from participant recruitment, with a well-designed number of participants, inclusion–exclusion criteria, and case–control matching thoroughly planned and complied with to reduce interpersonal confounders (58, 59). The selection of tissue samples and sample collection procedures must also be well designed and complied with during project execution, as divergences at this level can also influence results. Sample collection and processing prior to testing must also be considered in this standardization effort and mandatorily included in research reporting. Although the literature mentions that sample features are not the biggest confounders (59), this does not mean that it has no influence on results, and thus attention must be placed to this important technical aspect of every research study. All technical steps required for NGS and adjuvant techniques, such as metabolomics or others, also require standardization, which can be another big challenge due to the wide variety of available options, and possibly available prices, as well as NGS platforms and other instruments, which are also starting to diversify. Efforts need to be made by the scientific community to reduce these technical biases, as they also influence results and, if not properly addressed, can impair the advancement of knowledge. Some literature already assays this standardization (56, 57), but with the vertiginous acceleration of NGS technologies, an update on this matter is required to deliver an updated standard proposal. The inclusion of big data and machine learning experts to support microbiologists is also important to consider. Systematic computational methodologies, known as big data analysis, will allow a faster and more complete microbiome analysis that can help interpret more information than just the microbial compositions of a set of samples (65). Machine learning technology, by integrating phenotypic variables with gut microbiome in predictive models, can prominently increase the ability to predict health outcomes associated with microbiome features (66). Without this combination of disciplines, all efforts of trying to interpret the tremendously huge and different dimensions of a gut microbiome will remain quite far from its entire potential.

The International Human Microbiome Consortium (https://human-microbiome.org/) is already a reality that needs to be further supported and promoted to increment this full-of-potential field of research. Studies with adequate power and sample size, with a reproducible analysis approach, and integration of a multidisciplinary effort to interpret results and predict health outcomes are mandatorily needed for those who are confined to a very strict, socially and financially highly demanding GFD and seek alternatives to circumvent their condition. Advancing knowledge on the influence that gut microbiome may have in CeD onset and perpetuation may pave the way for gut microbiome modulation in future therapies. This review aimed to highlight the existing research on this subject and provide insights that may help guide future directions to enhance the applicability of obtained results in the future.
